# The Nordic long-term OCD treatment study (NordLOTS): rationale, design, and methods

**DOI:** 10.1186/1753-2000-7-41

**Published:** 2013-12-19

**Authors:** Per Hove Thomsen, Nor C Torp, Kitty Dahl, Karin Christensen, Inger Englyst, Karin H Melin, Judith B Nissen, Katja A Hybel, Robert Valderhaug, Bernhard Weidle, Gudmundur Skarphedinsson, Petra Lindheim von Bahr, Tord Ivarsson

**Affiliations:** 1 Department of Child and Adolescent Psychiatry, Aarhus University Hospital Risskov, Risskov, Denmark; 2 Centre for Child and Adolescent Mental Health, Eastern and Southern Norway (R-BUP), Oslo, Norway; 3 Department of Child and Adolescent Psychiatry, Queen Silvia’s Children’s Hospital, Sahlgrenska University Hospital, Gothenburg, Sweden; 4 Regional Centre for Child and Youth Mental Health and Child Welfare, Central Norway, Klostergata 46, 3rd floor, Trondheim 4391, Norway; 5 St. Olavs Hospital, BUP Klinik, Klostergate 44/46, Trondheim, Norway; 6 Centre for Child and Adolescent Psychiatry, Stockholm, Sweden

**Keywords:** Study design, Multisite study, Child and adolescents, Obsessive-compulsive disorder, Cognitive behavioural therapy, Stepped care design, Treatment outcome

## Abstract

**Background:**

This paper describes and discusses the methodology of the Nordic long-term OCD-treatment study (NordLOTS). The purpose of this effectiveness study was to study treatment outcome of CBT, to identify CBT non- or partial responders and to investigate whether an increased number of CBT-sessions or sertraline treatment gives the best outcome; to identify treatment refractory patients and to investigate the outcome of aripiprazole augmentation; to study the outcome over a three year period for each responder including the risk of relapse, and finally to study predictors, moderators and mediators of treatment response.

**Methods:**

Step 1 was an open and uncontrolled clinical trial with CBT, step 2 was a controlled, randomised non-blinded study of CBT non-responders from step 1. Patients were randomized to receive either sertraline plus CBT-support or continued and modified CBT. In step 3 patients who did not respond to either CBT or sertraline were treated with aripiprazole augmentation to sertraline.

**Conclusions:**

This multicenter trial covering three Scandinavian countries is going to be the largest CBT-study for paediatric OCD to date. It is not funded by industry and tries in the short and long-term to answer the question whether further CBT or SSRI is better in CBT non-responders.

## Background

Obsessive-compulsive disorder (OCD) affects up to 1 in 50 people [1,2], often has its onset in childhood or adolescence [2-5], is associated with severe dysfunction and psychiatric comorbidity in most cases [4,6-9], and often has a chronic course [10]. According to NICE-guidelines the ideal initial treatment in children and adolescents is cognitive behavioural therapy (CBT) alone or CBT and SSRI. CBT seems roughly to have about twice the effect size of SSRI-treatment [11-13], although results between studies have varied a lot. The reasons for this variation have not been clear but may be a consequence of sample characteristics, design issues, and differences with regard to the CBT given.

The clinical effectiveness and the stability of treatment gains after CBT are still to be established [14-16]. In addition, we still do not know to which extent CBT-manuals are transferrable to ordinary clinical settings. Valderhaug et al. showed this to be the case in one study, however this needs to be replicated in studies with more clinics in both specialized OCD clinics and general child psychiatric outpatient clinics [17].

Using an effectiveness study design our specific aims with the NordLOTS study were:

1. to identify and describe a large group of patients with moderate to severe OCD in the Scandinavian countries,

2. to treat patients with CBT with the commonly used number of CBT-sessions with exposure and response prevention (step 1) and to study treatment outcome,

3. to identify CBT non- or partial responders and investigate whether an increased number of CBT-sessions or sertraline treatment gives the best outcome (step 2),

4. to identify treatment refractory patients (to both CBT and sertraline) and investigate the outcome of aripiprazole augmentation (step 3),

5. to study the outcome over a three year period for each responder (in the three steps) including the risk of relapse, and

6. to study predictors, moderators and mediators of treatment response (symptomatic, psychosocial, molecular genetics, neuropsychological factors).

## Study design

### Stepped care design, with three steps

Step 1 was an open and uncontrolled clinical trial in which all patients received cognitive behavioural therapy in the form of exposure and response prevention (E/RP) using 14 treatment sessions. Patients were classified as treatment-responders or non-responders based on Children’s Yale-Brown Obsessive Compulsive Scale (CY-BOCS), scores of 15 or below or 16 or above respectively post treatment.

Step 2 was a controlled, randomized, non-blinded study of CBT non-responders from step 1. The patients were randomized to receive either sertraline plus CBT-support (in which patients were instructed to practice exposure tasks learned in step 1 outside the study sessions) or continued and modified, individualized CBT.

**Figure 1:**
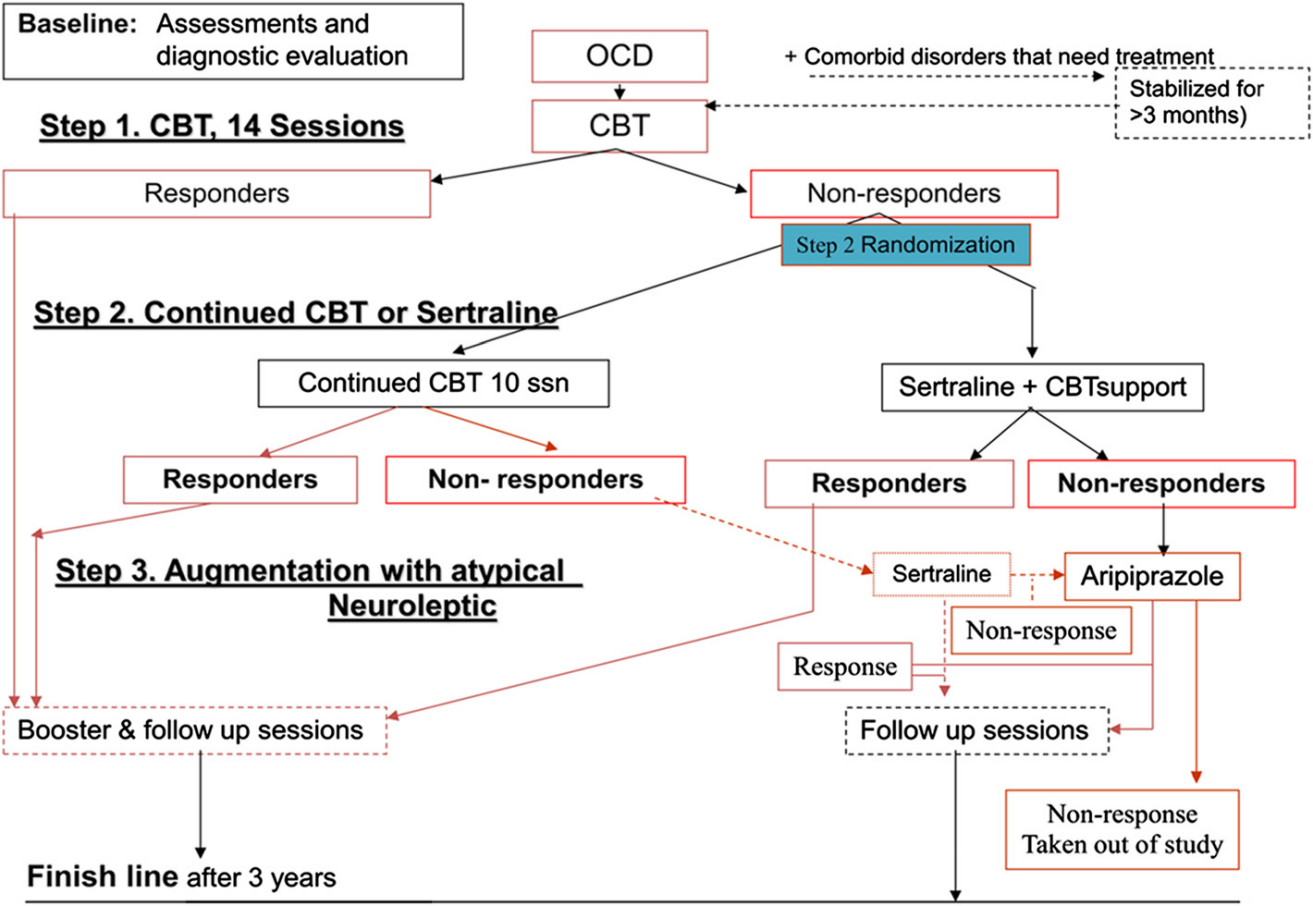
Flowchart.

In step 3 patients who did not respond to either CBT or sertraline were treated with aripiprazole augmentation to sertraline (Figure 1).

Randomization was performed using a stratified block method. Randomization sequence in an even ratio between both treatment modalities was done by generating in a two-stage process a block-wise randomization list. The randomization list was stratified according to gender and the presence or absence of tics/Tourette’s syndrome. Evaluation of treatment response was made by an independent evaluator that was not blind to the treatment allocation of each patient.

#### A multicenter trial

The Scandinavian countries included in this multisite trial (Sweden, Denmark, and Norway) represent a population of approximately 18 million people in total. Although there are some differences in terms of mutual intelligible languages we consider the Scandinavian countries to have mutual cultural background, to a certain extent common history, and therefore are to be considered as a rather homogenous population.

Included in the study trial were clinics which were specialized in OCD-treatment (Aarhus, Denmark, and Gothenburg, Sweden) and further centres in which OCD assessment and treatment were part of a general child and adolescent psychiatric units.

R-BUP in Oslo was the data-, hardware-, and coordination centre with the principal investigator (Ivarsson), Nordic coordinator (Dahl), and research assistant. Twice to thrice yearly meetings by the steering group (all authors) and the executive committee (the study’s initiators (Thomsen, Dahl, and Ivarsson) handled decision making. Yearly meetings at R-BUP with the clinicians and researchers were used to boost compliance.

At each site there was elected a local study coordinator who was responsible for recruitment to the study, the randomization procedure, and data entry.

A study visit was made at each site (once a year) by Tord Ivarsson in order to monitor randomization procedures, the handling of the instruments, assessment procedure, and the data entry into the Confirmit database.

Throughout the study period and during the planning of the study there were more meetings and telephone conferences between the presiding committee or the steering group.

## Study period

The study started the inclusion of patients in May 2008 and concluded June 2012.

### Population

We aimed at having broad inclusion criteria in order to design a study which was as close to daily clinical practise as possible. Of course, OCD should be the primary disorder and the exclusion criteria were merely those that would make the assessments unreliable or where other treatment needs would have higher priority.

#### Inclusion criteria

Children and adolescents were included into the NordLOTS on the basis of

1) primary diagnosis of OCD according to the criteria in the Diagnostic and Statistical Manual of Mental Disorders, Fourth Edition, Text Revision [18],

2) Children’s Yale-Brown Obsessive Compulsive Scale (CY-BOCS) entry score equal to or above 16,

3) ages 7 through 17,

4) patients with attention deficit hyperactivity disorder (ADHD) were included if they had been stable on medication for at least 3 months.

#### Exclusion criteria

Mental retardation (IQ below 70), disorders with higher treatment priority: autism, primary anorexia nervosa (anorexia in partial remission where OCD had become the residual and primary disorder was permitted), depression with suicidality that demanded CBT, SSRI treatment or inpatient treatment, psychosis, Asperger’s syndrome. However, PDD-NoS was allowed if CGI-score for the PDD was below or equal to 3 and CGI for the PDD-NoS < CGI for the OCD disorder, patients already under treatment for OCD with CBT, SSRI or atypical neuroleptics treatment within six months of study start, the patient or primary caregiver could not speak or understand the language in the country where the study was conducted.

For CBT non-responders who were randomised to sertraline in step 2, two additional exclusion criteria were applied: post pubertal girls with a positive pregnancy test, and post pubertal girls who were sexually active and who did not accept or tolerate adequate contraceptive methods.

#### Comorbidity

Patients with non-exclusionary comorbid disorder that could still be in need of a special treatment before entering the study, e.g. having ADHD, tics, Tourette’s syndrome or depression, were offered treatment for their comorbid disorder. Patients with treated comorbidity could be included.

## Assessment methods

### Measures

The following instruments were used as measures for inclusion, predictors and/or measures of treatment outcome (all measures were translated into Norwegian, Swedish, and Danish):

*Schedule for affective disorders and schizophrenia for school-age children – present and lifetime version (K-SADS-PL): The K-SADS-PL* is a semi-structured diagnostic interview that assesses a range of child psychopathology and demonstrates favourable psychometric properties [19]. K-SADS-PL has shown a good inter-rater reliability of 98% and a 1 to 5 week test-retest kappa of 0.80 for any anxiety disorder diagnosis [19]. Convergent and divergent validity have been documented in a Nordic sample of adolescents [20], moreover, the K-SADS have been used in previous OCD treatment trials [17,21]. Symptoms can be classified as “not present”, “possible”, “in remissions” or “certain”. In this study OCD diagnoses and comorbidity where based on symptoms classified as “certain” only.

*Children’s Yale-Brown Obsessive Compulsive Scale* (CY-BOCS): The CY-BOCS is a widely used, clinical-rated, semi-structured interview assessing the severity of OCD symptomatology [22]. The CY-BOCS records symptom categories and evaluates the severity of obsessions and compulsions using10 items, across five dimensions (time occupied by symptoms, interference, distress, resistance, and degree of control over symptoms). The total severity score range from 0 to 40. The CY-BOCS total score in range of 10–18, are considered mild, 19–28 moderate and scores from 29 and above severe [23]. CY-BOCS shows reasonable reliability and validity; with good to excellent inter-rater agreement [24,25]. A high internal consistency, 0.91, 0.68 and 0.84, for obsessions, compulsions and total score respectively, have been shown [26].

*Clinical Global Impressions-Severity* (CGI-S): Is a clinical rating of symptom severity. Ratings range from 0 (no illness) to 6 (extremely severe). The CGI-S correlates strongly with the CY-BOCS total score in paediatric OCD patients, and is widely used and has been shown to be treatment sensitive [25,27,28].

*Clinical Global Impressions-Improvement* (CGI–I): The CGI-I is used to assess overall clinical improvement based on symptoms observed and impairment reported using a seven point scale ranging from 0 (very much worse) to 6 (very much improved). The CGI-I scale was dichotomized so patients that received a rating of 5 (much improved) or 6 (very much improved) were collapsed in the analyses. The clinical-rated scale has been used successfully in patients with OCD [27,29].

*Children’s Global Assessment Scale* (CGAS): is a clinician’s rating on a numeric scale (1–100) of the patient’s overall level of functional strain [30]. The scale has shown good test-retest reliability (r = .62 and r = .76 with psychiatrist and staff respectively). Good inter-rater reliability as well [31]. Furthermore, it has demonstrated both discriminant and concurrent validity [30].

*Socioeconomic Status* (SES): We used Hollingshead’s two-factor index of social position to classify the socioeconomic position of each family [32]. This two-factor index combines ratings of parental occupation (1–9 scale) and parental education level (1–7 scale). Occupation is given a weight of 5 and education a weight of 3, this generates a summary score. The total scores were transformed into an ordinal scale that ranged between 1 and 5. SES was further dichotomized into two categories, high SES (scores 4–5) and low SES (scores 1–3).

*The Child Obsessive-Compulsive Impact Scale* (COIS-R): The COIS is a 33-item self-report questionnaire designed to assess the impact of OCD symptoms on the psychosocial functioning of children and adolescent in home, social and academic environment [1]. Both parent and youth versions are available. The patient and parents each rate how much difficulty the child have doing different everyday activities as a result of OCD. Each item is scored on a 4-point Likert scale (0 = not at all, 1 = just a little, 2 = pretty much, and 3 = very much). Both the child and parent versions have shown moderate to high internal consistency, for children α = 0.78 and parents α = 0.92 [1].

*Child Behavior Checklist* (CBCL): The CBCL is a 113-item parent-report form designed to assess a wide range of child behavioural and emotional problems. Parents rate items on a three-point scale (0 = not true; 1 = somewhat or sometimes true; and 2 = very or often true). This widely used index has established psychometric properties across a variety of clinical and non-clinical populations [33]. The CBCL has shown a mean test-retest reliability between 0.95-1.00 and internal consistency from α = 0.78 to α = 0.97 [33].

*Family Accommodation Scale* (FAS): The FAS is a 12 item clinician-rated instrument, designed to assess the family’s accommodation to the child’s OCD-symptoms during the previous month [34]. The FAS includes items that measures the extent to which family members provide reassurance or objects needed for compulsions, decreased behavioural expectations of the child, modify family activities or routines, or help the child avoid objects, places or experiences that cause distress. The FAS has demonstrated good psychometric properties including good internal consistency (α = 0.76 to α = 0.80) [34,35], and positive correlation with measures of OCD-symptoms severity [36] and family discord [34].

*Screen for Child Anxiety Related Emotional Disorders* (SCARED): The SCARED is a psychometrically sound child- and parent-report questionnaire which assesses the presence of DSM-IV anxiety symptoms [37,38]. SCARED total scores were used in these analyses. Scores range from 0 to 82 with higher scores indicating greater impairment and severity. The internal consistency of the SCARED total score was α = 0.94 [39].

*The Mood and Feelings Questionnaire* (MFQ): The MFQ is based on DSM-III-R criteria for depression and assesses the presence of depressive symptoms by means of 13 items [40]. Scores range from 0 to 26 with higher scores indicating greater impairment and severity. The MFQ has sound psychometric properties [41], and the MFQ total score has shown internal consistency of α = 0.75 to α = 0.78 [42].

*Family history* of OCD: during baseline assessment parents were asked, in a clinical interview, if they ever have been suffering from OCD. For the present study, a positive family history of OCD means that either the parent(s) and/or the siblings of the identified patient had been diagnosed with OCD.

*Parental psychopathology*: During baseline assessment parents were asked about psychological symptoms and diagnosed psychiatric problems (yes/no). For the present study, a positive history of parental psychopathology means that a parent(s) of the identified patient had been diagnosed with any psychiatric diagnosis.

*Autism Spectrum Screening Questionnaire* (ASSQ): The ASSQ was used for a dimensional measure of autism spectrum symptoms [43]. The internal consistency of the ASSQ total score was α = 0.86 [43].

*The EAS Temperament Questionnaire* (EAS): was used for a dimensional measure of temperament. The questionnaire is a parent report consisting of 20 Likert-scaled items relating to three subscales: emotionality, activity and sociability. The internal consistency of the EAS total score has shown to be α = 0.70 [44].

*Questionnaire for Measuring Health-Related Quality of Life in Children and Adolescents* (KINDL): was used as self-report questionnaire for children and adolescents as well as a proxy version completed by one of the parents to assess perceived quality of life [45]. The questionnaire consists of 24 items equally distributed into seven subscales. Mean item scores are calculated for all subscales and the total quality of life (QOL) scale, which are transformed to a 0–100 scale, 0 indicates very low and 100 very high QOL. The internal consistency for the children’s self-report total score was α = 0.82 [45].

*Five Minute Speech sample* (FMSS): The FMSS provides a measurement of parents’ Expressed Emotion (EE) toward their child [46]. The criteria for scoring EE from the FMSS were developed by Magaña et al. (1986) and are based on analyses of the affective quality of the total five minute monologue. Inter-rater reliability is assessed regularly in the laboratory, internal consistency range from α = 0.70 to α = 0.80 [47].

*Compliance*: During the treatment the clinician assessed the patients’ and the parents’ compliance to the therapy and in therapy. This assessment was done in sessions 2, 7, and 13. Compliance was assessed at a five point scale ranging from 0 (no compliance) to 4 (very good compliance).

*Credibility*: During treatment, in sessions 2, 7 and 13, the patients and the parents were given a form. They were asked to rate their credibility to the CBT-treatment, if they believed that the therapy would be helpful for them. Credibility was assessed at a five point scale ranging from 0 (no credibility to the therapy) to 4 (very much credibility).

### Treatments

#### CBT step 1

CBT step 1 involved E/RP based on the treatment manuals by March and Mulle as well as an adapted version by Piacentini (unpublished material, 1998), adding more family intervention. The manual was translated from English and adapted to fit Nordic conditions by a group of therapists from the three Nordic countries [48]. Only minor adaptations were necessary, mostly by revising the overall instructions and general descriptions of the main components of the treatment and by putting some more weight on the CBT triangle (the interrelation between thought-emotion-behaviour). Also, our manual put some more stress on the importance of the formulation of exact goals for the child’s play. Nevertheless, the main components from the manuals by March and Mulle, and by Piacentini, were kept unchanged.

An overview of the treatment sessions and the assessment procedures is presented in Table 1.

**Table 1:** Content of CBT-sessions and assessments

**Time**	**Assessment**	**Assessment instrument**	**Parents involved in**
0	Assessment by independent evaluator	K-SADS, CBCL, CY-BOCS, CGI, MFQ, ASSQ, COIS, CGAS, FAS, EAS, SCARED-R	Whole session
1	Psycho-education: Model for understanding and treatment	CGI-I, Compliance	Whole session
2	Externalising of OCD	CGI-I,	Whole session
3	Cognitive training and further assessment of OCD	CGI-I,	30 min
	Parents: *Negative attributions on OCD and the child*		
4	Test-exposure and tool box	CGI-I,	30 min
	Parents: *Parents’ role, guilt-feeling and self-reproach*		
5	E/RP; fight against OCD	CGI-I,	30 min
	Parents: *The family’s involvement in OCD*		
6	E/RP; get more control over OCD	CGI-I,	30 min
	Parents: *The child’s own responsibility for the treatment*		
7	E/RP; support the child or the OCD?	CGI-I, Compliance, CY-BOCS, CGAS, to bring home: MFQ, COIS	Whole session
	Joint hour with parents: *Repetition of parents’ role, milestones*		
8	E/RP; comorbidity and special therapeutic needs.	CGI-I,	30 min
	Parents: S*econdary winnings and other obstacles*		
9	E/RP; continue the fight against OCD	CGI-I,	30 min
	Parents: *Separate OCD from other problems*		
10	E/RP; continue the fight against OCD	CGI-I,	30 min
	Parents: *Unity and taking care of the family*		
11	E/RP; Going through the treatment session	CGI-I,	Whole session
	Parents: *Group-gathering – problem solving*		
12	E/RP; turning point	CGI-I,	30 min
	Parents: *How can parents prevent relapse*		
13	E/RP; prevent relapse	CGI-I, Compliance, CY-BOCS, CGAS.	30 min
	Parents: *What to do in case of relapse*	CBCL, MFQ, COIS, SCARED-R.	
	Check that date for independent evaluation is set		
14	Closing ceremony	CGI-I,	Whole session
	Getting together with the parents*: Going through the treatment process*		

All included patients should ideally have 14 sessions across 14 weeks. Breaks in CBT treatment were minimized and out of 14 sessions at least 10 were performed during at most 4½ months.

In doubtful cases the research group decided whether a patient was to be excluded due to fragmented CBT. Children who were early responders and who wanted to terminate treatment were encouraged to continue to 14 sessions, however, if not possible and fewer sessions had to be allowed (e.g. 1–7 sessions of CBT), ratings at 14 weeks post start should be fixed in time.

Patients who dropped out during step 1 or later were followed in an observational co-study of the NordLOTS using the same follow-up time points.

#### Step 2 CBT

For step 2, patients were randomized to either SSRI-treatment or CBT in a revised/reformulated version.

Patients randomized to continued CBT received 10 additional treatment sessions over 16 weeks. The same CBT-principles as used in step 1 were used in step 2. However, the therapist was allowed to take an individual approach to treatment based on reassessment of the patient, focusing on factors that may have led to inferior CBT-response. E/RP was adjusted to the problems encountered in step 1. Avoidance was also reassessed, and measures taken to minimize it. In this way the therapist was allowed to adapt CBT-manual to the individual child, situation, family expectations, etc. This was done in order to examine if a more individualized approach could make the CBT-treatment more effective.

#### Step 2: Medication

The treatment of sertraline included 6 sessions over 16 weeks (week 0, 2, 5, 8, 12, and 16). The pharmacotherapy treatment manual was adapted from the manual used in the POTS study [49]. A starting dose of 25 mg was titrated up to 100 mg over four weeks. Children below 10 years with low weight could be given a lower starting dose. If response was inadequate at a stable dose of 100 mg, after a minimum of 3 weeks, the dose was increased after serum concentration was controlled, if deemed necessary, up to a maximum 200 mg. Response and side effects were controlled at every visit, and dose reduced if necessary. The manual consisted of CBT-support where patients were instructed to practice exposure tasks learned in step 1 outside the study sessions. The main aims of the CBT support was to maintain treatment gains from the first step, to support an active fight against OCD-symptoms upholding a belief that the medication will help, to increase compliance and identify obstacles, and to ensure that medication is accompanied by the same psychological attitude in all cases. However, no new tasks were introduced.

Parents were involved at all medication visits, receiving feed-back about the child’s progress and treatment. While parents were encouraged to praise the child for resisting compulsions, other interventions directed at parents were prohibited during pharmacotherapy (Table 2).

**Table 2:** Assessments and dosing schedule in sertraline step 2

**Week**	**Dose (mg)**	**Range (mg)**	**Assessments**	**Actions**
				Check Adverse Events Scale (baseline) (AE), Somatic assessment (SA), Clinical Global Impression (CGI)
0	25 × 3 days, then 50	25-50	CY-BOCS incl. CGI (use CY-BOCS at step 1 session 13 if < 3 weeks, else reassessment in point 10c above), CGAS, blood pressure (BP), pulse, weight, length, side effects (AE)	AE, SA, CGI, Clinical Global Impression - Improvement (CGI-I)
2	75	50-75	CGI-I, CGI, BP, pulse, weight, length, side effects, treatment credibility	AE, SA, CGI, CGI-I
3-4	100	75-100	CGI-I, CGI, BP, pulse, weight, length, side effects	AE, SA, CGI, CGI-I, dose correction based on response
5-7	150	75-150	CGI-I, CGI, BP, pulse, weight, length, side effects, treatment credibility, KINDL (“independent rater”)	AE, SA, CGI, CGI-I, dose correction on response
8-12	200	75-200	CGI-I, CGI, BP, pulse, weight, length, side effects, treatment credibility	AE, SA, CGI, CGI-I, dose correction based on response
12-16	200	75-200	CY-BOCS incl. CGI/CGI-I, CGAS, Scared-R, MFQ, COIS, FAS, KINDL (“independent rater”), BP, pulse, weight, length, side effects, treatment credibility	AE, SA, CGI, CGI-I, dose correction based on response

#### Response to step 2 sertraline treatment

If assessments in session six (at 16 weeks) showed the patient to have a CY-BOCS score of 15 or below, the patient was considered a responder and went to follow-up including sertraline medical checkup and eventually sertraline treatment termination.

If assessment in session six at 16 weeks showed the patient to have a CY-BOCS score of 16 or above this patient was a non-responder and went to step 3 (see later).

Medical checkups and assessments took place every third month. As part of the follow-up (see later) assessments were performed after 6, 12, 24, and 36 months.

#### Maintenance doses of sertraline

Criteria for lower maintenance dose were checked at every visit: if the patient was very much improved (CGI-I = 6), if the OCD-illness was subclinical or in full remission (CGI-S = 0 or 1), if CY-BOCS scores are ≤ 10 points.

If lowered dose lead to worsened OCD or functioning, the dose was increased to full level again.

#### Criteria for sertraline termination

If a patient fulfilled termination criteria during medication follow-up, i.e. 6 months of subclinical OCD or full remission, sertraline was lowered with 25% every one to two weeks until a sertraline dose of 25 mg.

#### Step 3: Aripiprazole augmentation to sertraline in CBT + sertraline non-responders

Patients who were non-responders or partial responders within step 2 of the NordLOTS-study were asked to participate in step 3. Step 3 was based on augmenting sertraline treatment with the antipsychotic drug aripiprazole. Thus, all patients in step 3 were given both sertraline and aripiprazole.

Patients who respond to this regime (i.e. CY-BOCS ≤ 15 and CGI-S ≤ 2, and CGI-I ≥ 5) are followed for the total three year period.

#### Follow-up

All included patients are being followed-up at 6, 12, 24, and 36 months. They will be assessed with the instruments described in Table 3.

**Table 3:** Overview of measurements

		**Step 1**		**Step 2, SSRI and CBT**			**Follow up (months)**			
**Weeks**	**Baseline**	**7 wks**	**13**	**Reassess**	**8**	**16**	**6**	**12**	**24**	**36**
History	Yes									
K-SADS [19]	Yes			Possible						
CY-BOCS [20]	Yes	Yes	Yes	Yes	Yes	Yes	Yes	Yes	Yes	Yes
CGI-S/CGI-I [21]	Yes	Yes	Yes	Yes	Yes	Yes	Yes	Yes	Yes	Yes
COIS [22]	Yes		Yes	Yes		Yes	Yes	Yes	Yes	Yes
CGAS [23]	Yes		Yes	Yes		Yes	Yes	Yes	Yes	Yes
CBCL [24]	Yes		Yes			Yes	Yes	Yes	Yes	Yes
YSR [24]	Yes		Yes				Yes	Yes	Yes	Yes
FAS [25]	Yes		Yes		Yes	Yes	Yes	Yes	Yes	Yes
FES [14]	Yes		Yes		Yes	Yes	Yes	Yes	Yes	Yes
PABS [15]	Yes		Yes		Yes	Yes	Yes	Yes	Yes	Yes
MFQ	Yes		Yes			Yes				
SCARED	Yes		Yes			Yes				

### Genetics

#### Concomitant study of genetics and heredity

All patients included in the study were asked to participate in a concomitant study on genetic aspects of OCD. All patients filled in a heredity scheme which was used in a semi-structured format quering about relatives that had any of the specified disorders and their severity. This entails taking a saliva sample from the patient and both parents for the study of candidate genes that are part of the glutamate system using a trios design. Treatment response will, we propose, be influenced by genetics as well as by other factors that might interact with genetic factors. Probably, different genes might predict CBT-response as compared to sertraline response.

### Quality assurance

All therapists were certified psychotherapists trained in CBT with a special approach to OCD including seminars with local and international speakers training in OCD [1,50]. Before being accepted as a study therapist, each therapist had to have at least two patients with completed CBT-manual.

During the study there was ongoing training in the form of mutual seminars for all study sites and all therapists (at least once a year). Furthermore, the therapists received supervision at each site, and each study patient was discussed at the clinical conferences in the units.

All together, 35 therapists carried out the CBT in step 1. 11 therapists only had two completed patients, six therapists had three patients, and the total range of patients per therapist was 2 to 40. 14 therapists came from Swedish centres, 17 from Norwegian centres and four from the Danish centre.

### Treatment fidelity

Each therapist had to fill in a checklist after each session whether he/she had performed E/RP, home work, psychoeducation etc. (different items from session to session according to the treatment manual). Sessions were audio taped so that all phases of CBT (psychoeducation, E/RP and the termination phase) were included.

Three categories of treatment fidelity were evaluated: manual competence, relationship competence and overall evaluation of the session. Scores in each of these categories ranged from 1 = very bad compliance to 4 = very good compliance. Experienced therapists from each country scored recordings of the therapist’s competences using audio tapes and the NordLOTS Treatment Integrity Scale (TIS) for all 14 sessions.

The results of this are described in Torp et al., yet unpublished material.

### Reliability of independent evaluator ratings

Each site had independent evaluators. The evaluators from all sites were jointly trained to reliable standard of the CY-BOCS and the Kiddie-SADS through joint interviews, videotaped reviews, and discussion. To maintain and assess reliability between sites 15% of audio taped interviews were chosen for a blind review to assess inter-rater and cross-site reliability. Independent evaluators were used for CY-BOCS at baseline and weeks 7 and 13. Intra-class correlation coefficients of inter-rater agreement were: obsessions ICC = 0.94 (95% CI 0.85-0.97), compulsions ICC = 0.87 (95% CI 0.67-0.93) and total score ICC = 0.92 (95% CI 0.78-0.97).

### Statistical aspects

#### Sample size and randomization

The total number of patients included in step 1 was defined according to the power requirements for step 2.

The primary criteria for entry in step 1 and 2 were moderate to severe symptoms.

Remission in our study was defined as those who had CY-BOCS lower than 16 on the week 13 assessment. Those patients can be regarded to have mild OCD [51,52]. As step 2 was a randomized, controlled trial that included drug treatment we regarded patients with mild OCD (CY-BOCS < 16) to be unsuitable to include. In step 2, we estimated a remission rate of 75% for sertraline plus CBT-support but 50% for the continued CBT without medication. Thus, 58 individuals in each treatment group had to be included to detect such a difference in proportions with an α of 0.05 and β of 0.8 [53]. Seven patients in each treatment condition did not complete the trial and refused any further evaluation on outcome measures.

For analyzing moderators and binary outcome data, multiple imputations were used to replace missing values. This was done with a sequential regression multivariate imputation algorithm with the aid of the IVEware package for SAS [54,55]. The model of imputation included all outcome measures, time in weeks, treatment indicators, stratification variables (sex and tic disorder) and all possible predictors and moderators. A total of 200 data sets were generated to maximize variance [56]. Results that are reported were calculated using the Rubin rules for combining the results of the 200 identical analyses [54].

All randomized patients with a CY-BOCS score more than or equal to 16 before treatment started were included in the analyses according to the principles of intent-to-treat. Analyses were performed with the SAS Statistical Software, version 9.3. The CY-BOCS total score at each assessment point, including step 1, was analyzed with a maximum likelihood estimation of multilevel models [57]. The model included fixed effect days from baseline, introduction of step 2 treatment and days from randomization. Random effects were intercept and days from baseline. The models were fit by using the PROC MIXED in SAS Statistical Software, version 9.3 [58].

Multivariate χ2 test was conducted for testing of between-group differences in the response at week 16. This was done for all the 200 imputations and the results were combined and reported as an F-statistics with the help of the SAS macro combchi [59].

### Study monitoring

Independent study monitoring was performed by Dr. Zeiner who was neither involved in the NordLOTS study group nor in the NordLOTS steering group. He was in charge of monitoring step 2 of the trial for signs of ^1)^ safety, ^2)^ effectiveness, and ^3)^ futility. Scientific advisors were Professor John March, Associate Professor Martin Franklin, Professor Bo Larsson, and Professor John Piacentini.

## Discussion

### Alternative designs to step 1

In this paper we describe the rationale behind the chosen design for the NordLOTS study. Instead of our design we could have chosen CBT versus SSRI, or CBT versus SSRI + CBT. However, both research questions have been addressed in previous studies [28,60,61].

Previous studies have shown that CBT is as sufficient as or even more efficient than SSRI in treating moderate to severe OCD in children and adolescents (Ivarsson et al., yet unpublished material). In the POTS-study it has been shown that CBT alone, sertraline alone and combined treatment are all significantly more efficient than placebo [28]. The POTS-study also documented that combined treatment proved superior to CBT alone and to sertraline alone. However, the remission rate for combined treatment did not differ from that for CBT alone but from sertraline alone and placebo. These studies have led to the general suggestion in most guidelines that children and adolescents with OCD should begin treatment with a combination of CBT plus a SSRI or CBT alone.

The reason for choosing an open step 1 in which all patients received CBT (efficiency study) was to pursue the idea of a stepped care model.

We, therefore, chose CBT as treatment for all enrolled in the study, step 1. Why did we choose 14 sessions? Most of the studies of CBT-outcome in paediatric OCD have employed similar protocols involving weekly treatment over 12–14 weeks [62-64]. Other studies have investigated the effectiveness of 14 weekly sessions over 12 weeks or 18 sessions over 4 weeks without finding any difference. We do not know whether the chosen number of CBT-sessions is indeed the optimal treatment duration. In a meta-analysis of Olatunji et al., 2013, the number of sessions was not related to CBT effect sizes. However, the analysis included all patients no matter whether they were treatment completers or partial- or non-responders [65]. In adults no difference has been found at follow-up between intensive or twice weekly CBT.

The POTS-II study investigated the difference in efficacy between medication, medication plus 7 CBT-sessions, and medication plus 14 CBT-sessions and concluded that dissemination of full CBT-augmentation for paediatric OCD partial responders of SSRI should be an important public health objective [63]. Compared to POTS-II we chose a design which goes in the opposite direction offering CBT as a first step and eventually randomization to SSRI/continued CBT as a step 2, and augmentation to medication therapy as a step 3.

The components of CBT for children have often been poorly specified [66]. E/RP and anxiety management have been used in most studies. It has been shown that E/RP alone (with minimal anxiety management) was sufficient to achieve significant benefit. Other studies have stressed the importance of cognitive techniques [15,28,66]. Few studies have compared CBT in group format and found the same effect of CBT as individualized treatment [14,60]. In few studies the involvement of parents have been tested [14,67]. In this study we chose the protocol by Foa et al. which was translated and adapted to fit Nordic CBT-therapists [48].

### Alternatives to step 2 design

In our study non- or partial responders from step 1 were further randomised to a controlled study with a reformulated, intensified CBT or SSRI (+ CBT-support as defined). We chose this design in order to specifically answer the question: does a continued (based on case formulation) CBT help non- or partial responders after 14 sessions of manualized CBT as much as treating with SSRI? This is an often asked question in daily clinical practice.

Why prolonged CBT in step 2? – Some researchers, and many clinicians, have argued for longer treatment in cases of non-response to 14 sessions of CBT [61]. There might be a need for more hours due to the severity of the disorder, and there might be patient characteristics that have led to a delayed CBT-response. Therefore, we have chosen that continued CBT might be a better and more exacting test to pit against SSRI in CBT initial non-responders than would be for instance placebo. The backside of our choice is that the design does not permit to separate out late effects from step 1 CBT.

How important is CBT-support during medical treatment? As we wanted a clinically meaningful treatment arm with SSRI, also including the benefits from the CBT in step 1, we allowed CBT-support - defined as support from the therapist aiming to maintain the gains from the manualized CBT. However, to be able to compare with the CBT step 2-arm no E/RP should be implemented by the pharmacotherapist, as well as any psychotherapy or cognitive behavioural or family intervention during the 30 week study period. Moreover, having all pharmacotherapists give reasonably identical interventions with regard to the OCD-symptoms ought to reduce chance variance due to the pharmacotherapist’s attitude to and knowledge about CBT.

### Discussion of representativeness of the sample

The inclusion and exclusion criteria would indicate that all patients with OCD in the study would be fairly representative of patients within the clinics. Our exclusion criteria concern only a small group of OCD-patients and a few patients with higher treatment priorities.

### Outcome measures

A cut-off score of 16 or more on the CY-BOCS has been used in previous treatment studies including a number of pharmacological studies [68-70]. In addition, a continuous measurement such as a 30% reduction on the CY-BOCS score is clinically meaningful in order to capture differences in OCD severity and in order to specifically look at subgroups with different responses to treatment within the area of severe, moderate, and mild OCD.

The definition of 10 as a cut-off score for clinical remission has been used in previous studies [28], however, it has recently been questioned by Storch et al. [71]. Response to treatment may be defined differently via a wide range of CY-BOCS percent reductions. Riddle et al. has defined treatment response as a reduction of 25% whereas Geller et al. used a 40% reduction [62]. Tolin aimed at determining the optimal percent reduction cut-offs on the Y-BOCS in 87 adults with OCD after receiving CBT [72]. A Y-BOCS reduction of 30% optimally predicted treatment response, and a Y-BOCS reduction of 40–50% optimally predicted remission. Storch et al. (2010) replicated this analysis in 109 adolescents with OCD. They found that maximally efficient CY-BOCS cut-off was observed at 25% reduction for treatment response, and a 45–50% reduction for symptom remission and that a CY-BOCS score of 14 or below best reflected remission after treatment.

### Importance of the study

This is a large study examining the efficiency of CBT in patients in the Scandinavian countries based on a manualized CBT-programme and is to date the largest study in the World. It is not funded by industry and tries in the short and long-term to answer the question whether further CBT or SSRI is better in CBT-non-responders. Knowledge of *real world effectiveness* is needed for the plans of health organizations, for therapists and doctors who consider choice of treatment, and for the informed patient or parent who wants to participate in treatment planning. Expert clinical guidelines need to be tested empirically to be the basis of these health decisions. The units in the NordLOTS ranges from university based specialized OCD-clinics to unspecialized child and adolescent psychiatric outpatient units. This will make it possible to study the contribution of the type of clinic to treatments success as well.

### Relevance to practitioners

First of all, the NordLOTS leads to the development of a CBT and psychopharmacological treatment manual for all Scandinavian countries.

The NordLOTS will hopefully be able to guide practitioners with patients who do not respond to CBT. Furthermore, practitioners are responsible for patients for a long time – in some cases across childhood and adolescence. Most studies only give information on the efficacy of methods across a short period of time. However, the practitioner with a long-term commitment to his/her patient has both to initiate and terminate treatment. The NordLOTS hopefully will be able to handle some but not all of these hurdles. It will give valid information on long-term perspective on treatment up to three years. It will then provide some answers on how to plan treatment with a longitudinal perspective.

### Perspectives of the NordLOTS

The studies of psychosocial, symptomatic, and genetic factors on the treatment study will increase our understanding of OCD. In view of the high risk of chronic impairment and suffering in paediatric OCD these aspects need to be highlighted [73,74].

The spreading of the research protocol across the Nordic countries, the therapeutic experience gained from this long study will help us in spreading the expertise in CBT and the adequate use of medication to a high number of outpatient clinics also outside the university based child and adolescent psychiatry.

### Ethics

The study was approved by the National ethical Committees in Denmark, Norway, and Sweden. The study was approved by all data authorities in all three countries. Oral and written information was given, and written consent from the parents and patients was received for the RCT-part of the study.

#### Reporting of results

Results from the CBT-step 1 and predictors for treatment success are going to be published (Torp et al., yet unpublished data). Results from step 2, the randomised trial of continued CBT versus SSRI treatment will be reported (Skarphedinsson et al., yet unpublished data). Data collection for six, 18, 24, and 36 months follow-up studies is ongoing.

EudraCT-number/trial number is 2009-011115-20.

### Key points

•The radionale and design of the NordLOTS study is described.

•NordLOTS is the largest multisite, multinational CBT-study for childhood and adolescent OCD describing the effectiveness of a manualized CBT-protocol.

•NordLOTS uses a stepped care design and will attempt to answer the question: What is best for non-responders to 14 sessions of CBT: SSRI-treatment or continued CBT?

•NordLOTS is not sponsored by industry

•NordLOTS will describe the short term as well as the long-term effect of CBT and/or SSRI-treatment after 1, 2, and 3 years.

## Competing interests

The authors declare that they have no competing interests.

## Authors’ contributions

All authors have participated in the design of the study and in the collection of data. PHT was in charge of writing this paper on the design and rationale, GS and NCT were in charge of the statistical design. IE, KC, KD, BW, RV, NCT, and KHM all took responsibility in creating the Nordic CBT-manual and also in supervising therapists. All authors read and approved the final manuscript. PHT, KD, TI, and BW were members of the NordLOTS steering group.

## References

[B1] Piacentini J Peris TS Bergman RL Chang S Jaffer M Functional impairment in childhood OCD: development and psychometrics properties of the Child Obsessive-Compulsive Impact Scale-Revised (COIS-R) J Clin Child Adolesc Psychol 2007 7 645 653 10.1080/15374410701662790 18088221

[B2] Heyman I Fombonne E Simmons H Ford T Meltzer H Goodman R Prevalence of obsessive-compulsive disorder in the British nationwide survey of child mental health Br J Psychiatry 2001 7 324 329 10.1192/bjp.179.4.324 11581112

[B3] Piacentini J Bergman RL Obsessive-compulsive disorder in children Psychiatr Clin North Am 2000 7 519 533 10.1016/S0193-953X(05)70178-7 10986725

[B4] Valderhaug R Ivarsson T Functional impairment in clinical samples of Norwegian ans Swedish children and adolescents with obsessive-compulsive disorder Eur Child Adolesc Psychiatry 2005 7 164 173 10.1007/s00787-005-0456-9 15959662

[B5] Flament MF Cohen D Maj M, Sartorius N, Okasha A, Zohar J Child and adolescent obsessive-compulsive disorder: a review Obsessive-Compulsive Disorder 2000 Chichester: John Wiley & Sons, Ltd 147 183

[B6] Piacentini J Bergman RL Keller M McCracken JT Functional impairment in children and adolescents with obsessive-compulsive disorder J Child Adolesc Psychopharmacol 2003 7 S53 S60 12880501 10.1089/104454603322126359

[B7] Sørensen CB Kirkeby L Thomsen PH Quality of life with OCD. A self-reported survey among members of the Danish OCD Association Nord J Psychiatry 2004 7 231 236 10.1080/08039480410006287 15204211

[B8] Swedo SE Rapoport JL Leonard HL Lenane M Cheslow DL Obsessive-compulsive disorder in children and adolescents: clinical phenomenology of 70 consecutive cases Arch Gen Psychiatry 1989 7 335 341 10.1001/archpsyc.1989.01810040041007 2930330

[B9] Ivarsson T Melin K Wallin L Categorical and dimensional aspects of co-morbidity in obsessive-compulsive disorder (OCD) Eur Child Adolesc Psychiatry 2008 7 20 31 10.1007/s00787-007-0626-z 18004647

[B10] Eisen JL Pinto A Mancebo MC Dyck IR Orlando ME Rasmusen SA A 2-year prospective follow-up of the course of obsessive-compulsive disorder J Clin Psychiatry 2010 7 1033 1039 10.4088/JCP.08m04806blu 20797381 PMC4083757

[B11] Geller DA Biederman J Stewart SE Mullin B Martin A Spencer T Which SSRI? A meta-analysis of pharmacotherapy trials in pediatric obsessive-compulsive disorder Am J Psychiatry 2003 7 1919 1928 10.1176/appi.ajp.160.11.1919 14594734

[B12] March JS Mulle K Herbel B Behavioral psychotherapy for children and adolescents with obsessive-compulsive disorder: an open trial of a new protocol-driven treatment package JAMA 1994 7 333 341 10.1097/00004583-199403000-00006 8169177

[B13] Watson HJ Rees CS Meta-analysis of randomized, controlled treatment trials for pediatric obsessive-compulsive disorder J Child Psychol Psychiatry 2008 7 489 498 10.1111/j.1469-7610.2007.01875.x 18400058

[B14] Barrett PM Healy-Farrell L March JS Cognitive-behavioral family treatment of childhood obsessive-compulsive disorder: a controlled trial J Am Acad Child Adolesc Psychiatry 2004 7 46 62 10.1097/00004583-200401000-00014 14691360

[B15] Abramowitz JS Whiteside SP Deacon BJ The effectiveness of treatment for pediatric obsessive-compulsive disorder: a meta-analysis Behav Ther 2005 7 55 63 10.1016/S0005-7894(05)80054-1

[B16] Turner CM Cognitive-behavioural theory and therapy for obsessive-compulsive disorder in children and adolescents: current status and future directions Clin Psychol Rev 2006 7 912 938 10.1016/j.cpr.2005.10.004 16624461

[B17] Valderhaug R Larsson B Götestam KG Piacentini J An open clinical trial of cognitive-behaviour therapy in children and adolescents with obsessive-compulsive disorder administered in regular outpatient clinics Behav Res Ther 2007 7 577 589 10.1016/j.brat.2006.04.011 16836977

[B18] American Psychiatric Association Diagnostic and statistical manual of mental disorders, fourth edition, text revision (DSM-IV-TR) 2000 Washington, DC: American Psychiatric Association

[B19] Kaufman J Birmaher B Brent DA Rao U Flynn C Moreci P Schedule for Affective Disorders and Schizophrenia for School-Age Children-Present and Lifetime Version (K-SADS-PL): initial reliability and validity data J Am Acad Child Adolesc Psychiatry 1997 7 980 988 10.1097/00004583-199707000-00021 9204677

[B20] Lauth B Arnkelsson GB Magnússon P Skarphédinsson GÁ Ferrari P Pétursson H Validity of K-SADS-PL (Schedule for Affective Disorders and Schizophrenia for School-Age Children - Present and Lifetime Version) depression diagnoses in an adolescent clinical population Nord J Psychiatry 2010 7 409 420 10.3109/08039481003777484 20438289

[B21] Freeman JB Garcia AM Coyne L Ale C Prezeworski A Himle M Early childhood ocd: preliminary findings from a family-based cognitive-behavioral approach J Am Acad Child Adolesc Psychiatry 2008 7 593 602 10.1097/CHI.0b013e31816765f9 18356758 PMC2820297

[B22] Goodman WK Price LH Rasmusen SA Mazure C Fleischman RL Hill CL The Yale-Brown Obsessive Compulsive Scale. I. Development, Use, and Reliability Arch Gen Psychiatry 1989 7 1006 1011 10.1001/archpsyc.1989.01810110048007 2684084

[B23] March JS Biederman J Wolkow R Safferman A Mardekian J Cook EH Sertraline in children and adolescents with obsessive-compulsive disorder: a multicenter randomized controlled trial JAMA 1998 7 1752 1756 10.1001/jama.280.20.1752 9842950

[B24] Gallant J Storch EA Merlo LJ Ricketts ED Geffken GR Goodman WK Convergent and discriminant validity of the Children's Yale-Brown Obsessive Compulsive Scale-Symptom Checklist J Anxiety Disord 2008 7 1369 1376 10.1016/j.janxdis.2008.01.017 18329843

[B25] Storch EA Murphy TK Geffken GR Soto O Sajid M Allen P Psychometric evaluation of the Children's Yale-Brown Obsessive-Compulsive Scale Psychiatry Res 2004 7 91 98 10.1016/j.psychres.2004.06.009 15572188

[B26] Scahill L Riddle MA McSwiggin-Hardin M Ort SI King RA Goodman WK Children's Yale-Brown Obsessive compulsive scale: reliability and validity J Am Acad Child Adolesc Psychiatry 1997 7 844 852 10.1097/00004583-199706000-00023 9183141

[B27] Busner J Targum SD The clinical global impressions scale. Applying a research tool in clinical practice Psychiatry 2007 7 28 37 20526405 PMC2880930

[B28] Pediatric OCD Treatment Study (POTS) Team Cognitive-behavior therapy, sertraline, and their combination for children and adolescents with obsessive-compulsive disorder. The Pediatric OCD Treatment Study (POTS) randomized controlled trial JAMA 2004 7 1969 1976 15507582 10.1001/jama.292.16.1969

[B29] Garvey MA Perlmutter SJ Allen AJ Hamburger S Lougee L Leonard HL A pilot study of penicillin prophylaxis for neuropsychiatric exacerbations triggered by streptococcal infections Soc Biol Psychiatry 1999 7 1564 1571 10.1016/S0006-3223(99)00020-7 10376116

[B30] Shaffer D Gould MS Brasic J Ambrosini P Fisher P Bird H A Children's Global Assessment Scale (CGAS) Arch Gen Psychiatry 1983 7 1228 1231 10.1001/archpsyc.1983.01790100074010 6639293

[B31] Green B Shirk S Hanze D Wanstrath J The children's global assessment scale in clinical practice: an empirical evaluation J Am Acad Child Adolesc Psychiatry 1994 7 1158 1164 10.1097/00004583-199410000-00011 7982866

[B32] Hollingshead AB Two factor index of social position 1957 New Haven, CT Privately printed

[B33] Achenbach TM Maurish ME Child behavior checklist and related instruments The use of psychological testing for treatment planning and outcome assessment 1994 Hillsdale, NJ: Lawrence Erlbaum Associates 517 549

[B34] Calvocoressi L Mazure CM Kasl SV Skolnick J Fisk D Vegso SJ Family accommodation of obsessive-compulsive symptoms: instrument development and assessment of family behavior J Nerv Ment Dis 1999 7 636 642 10.1097/00005053-199910000-00008 10535658

[B35] Geffken GR Storch EA Duke DC Monaco L Lewin AB Goodman WK Hope and coping in family members of patients with obsessive-compulsive disorder J Anxiety Disord 2006 7 614 629 10.1016/j.janxdis.2005.07.001 16084686

[B36] Storch EA Geffken GR Merlo LJ Jacob ML Murphy TK Goodman WK Family accomodation in pediatric obsessive-compulsive disorder J Clin Child Adolesc Psychol 2007 7 207 216 10.1080/15374410701277929 17484693

[B37] Birmaher B Brent DA Chiapetta L Bridge J Monga S Baugher M Psychometric Properties of the Screen for Child Anxiety Related Emotional Disorders (SCARED): a replication study J Am Acad Child Adolesc Psychiatry 1999 7 1230 1236 10.1097/00004583-199910000-00011 10517055

[B38] Birmaher B Khetarpal S Brent D Cully M Balach L Kaufman J The Screen for Child Anxiety Related Emotional Disorders (SCARED): scale construction and psychometric characteristics J Am Acad Child Adolesc Psychiatry 1997 7 545 553 10.1097/00004583-199704000-00018 9100430

[B39] Muris P Mayer B Bartelds E Tierney S Bogie N The revised version of the Screen for Child Anxiety Related Emotional Disorders (SCARED-R): treatment sensitivity in an early intervention trial for childhood anxiety disorders Br J Clin Psychol 2001 7 323 336 10.1348/014466501163724 11593959

[B40] Angold A Costello EJ Messer SC Pickles A Development of a short questionnaire for use in epidemiological studies of depression in children and adolescents Int J Methods Psychiatr Res 1995 7 237 249

[B41] Messer SC Angold A Costello EJ Loeber R van Kammen W Stouthamer-Loeber M Development of a short questionnaire for use in epidemiological studies of depression in children and adolescents: factor composition and structure across development Int J Methods Psychiatr Res 1995 7 251 262

[B42] Wood A Kroll L Moore A Harrington R Properties of the mood and feelings questionnaire in adolescent psychiatric outpatients: a research note J Child Psychol Psychiatry 1995 7 327 334 10.1111/j.1469-7610.1995.tb01828.x 7759594

[B43] Ehlers S Gillberg C The epidemiology of Asperger syndrome. A total population study J Child Psychol Psychiatry 1993 7 1327 1350 10.1111/j.1469-7610.1993.tb02094.x 8294522

[B44] Mathiesen KS Tambs K The EAS temperament questionnaire - Factor structure, age trends, reliability, and stability in a Norwegian sample J Child Psychol Psychiatry 1999 7 431 439 10.1111/1469-7610.00460 10190344

[B45] Bullinger M Brutt AL Erhart M Ravens-Sieberer U BELLA Study Group Psychometric properties of the KINDL-R questionnaire: results of the BELLA study Eur Child Adolesc Psychiatry 2008 7 125 132 10.1007/s00787-008-1014-z 19132312

[B46] Magaña AB Goldstein MJ Karno M Miklowitz DJ Jenkins J Falloon IRH A brief method for assessing expressed emotions in relatives of psychiatric patients Psychiatry Res 1986 7 203 212 10.1016/0165-1781(86)90049-1 3704028

[B47] Asarnow JR Goldstein MJ Tompson MC Guthrie D One-Year Outcomes of Depressive Disorders in Child Psychiatric Inpatients: Evaluation of the Prognostic Power of a Brief Measure of Expressed Emotion J Child Psychol Psychiatry 1993 7 129 137 10.1111/j.1469-7610.1993.tb00975.x 8444988

[B48] Foa EB Kozak MJ Mastery of Obsessive-Compulsive Disorder: A Cognitive-Behavioral Approach Therapist Guide 1997 New York, NY: Oxford University Press, Inc.

[B49] Freeman JB Choate-Summers ML Garcia AM Moore PS Sapyta JJ Khanna MS The pediatric obsessive-compulsive disorder treatment study II: rationale, design and methods Child Adolesc Psychiatry Ment Health 2009 7 1 4 10.1186/1753-2000-3-1 19183470 PMC2646688

[B50] Franklin ME Kozak MJ Cashman LA Coles ME Rheingold AA Foa EB Cognitive-behavioral treatment of pediatric obsessive-compulsive disorder: an open clinical trial J Am Acad Child Adolesc Psychiatry 1998 7 412 419 10.1097/00004583-199804000-00019 9549962

[B51] Pallanti S Hollander E Bienstock C Koran LM Leckman JF Marazziti D Treatment non-response in OCD: methodological issues and operational definitions Int J Neuropsychopharmacol 2002 7 181 191 12135542 10.1017/S1461145702002900

[B52] Simpson HB Huppert JD Petkova E Foa EB Liebowitz MR Response versus remission in obsessive-compulsive disorder J Clin Psychiatry 2006 7 269 276 10.4088/JCP.v67n0214 16566623

[B53] Haynes R Sackett D Clinical epidemiology: how to do clinical practice research 2006 UK: Lippincott, Williams & Wilkins

[B54] Rubin DB Multiple Imputation for Nonresponse in Surveys 2004 Wiley Inter-Science: New York, NY

[B55] Raghunathan TE Lepkowski JM van Hoewyk J Solenberger P A multivariate technique for multiple imputing missing values using a sequence of regression models Surv Methodol 2001 7 85 96

[B56] Graham JW Olchowski AE Gilreath TD How many imputations are really needed? Some practical clarifications of multiple imputation theory Prev Sci 2007 7 206 213 10.1007/s11121-007-0070-9 17549635

[B57] Singer JD Willett JB Applied Longitudinal Data Analysis: Modeling change and event occurrence 2003 Oxford: Oxford University Press

[B58] Littell RC Milliken GA Stroup WW Wolfinger RD SAS System for Mixed Models 1996 Cary, NC: SAS Institute, Inc.

[B59] Allison PD Macro combchi 2013 Ref Type: Computer Program

[B60] Asbahr FR Castillo AR Ito LM Latorre MRDO Moreira MN Lotufo-Neto F Group cognitive-behavioral therapy versus sertraline for the treatment of children and adolescents with obsessive-compulsive disorder J Am Acad Child Adolesc Psychiatry 2005 7 1128 1136 10.1097/01.chi.0000177324.40005.6f 16239861

[B61] de Haan E Hoogduin KAL Buitelaar JK Keijsers GPJ Behavior therapy versus clomipramine for the treatment of obsessive-compulsive disorder in children and adolescents J Am Acad Child Adolesc Psychiatry 1998 7 1022 1029 10.1097/00004583-199810000-00011 9785713

[B62] Geller DA March JS Walter HJ Bukstein O Benson RS Chrisman A Practice parameter for the assessment and treatment of children and adolescents with obsessive-compulsive disorder J Am Acad Child Adolesc Psychiatry 2012 7 98 113 10.1016/j.jaac.2011.09.019 22176943

[B63] Franklin ME Sapyta JJ Freeman JB Khanna M Compton SN Almirall D Cognitive behavior therapy augmentation of pharmacotherapy in pediatric obsessive-compulsive disorder: the Pediatric OCD Treatment Study II (POTS II) randomized controlled trial JAMA 2011 7 1224 1232 10.1001/jama.2011.1344 21934055 PMC3495326

[B64] O'Kearney RT Anstey K von Sanden C Hunt A Behavioural and cognitive behavioural therapy for obsessive compulsive disorder in children and adolescents Cochrane Libr 20-1-2010. Ref Type: Electronic Citation 10.1002/14651858.CD004856.pub2 PMC8855344 17054218

[B65] Olatunji BO Davis ML Powers MB Smits JAJ COgnitive-behavioral therapy for obsessive-compulsive disorder: a meta-analysis of treatment outcome and moderators J Psychiatr Res 2013 7 33 41 10.1016/j.jpsychires.2012.08.020 22999486

[B66] Williams TI Salkovskis PM Forrester L Turner S White H Allsopp MA A randomised controlled tiral of cognitive behavioural treatment for obsessive compulsive disorder in children and adolescents Eur Child Adolesc Psychiatry 2010 7 449 456 10.1007/s00787-009-0077-9 19921305

[B67] Bolton D Perrin S Evaluation of exposure with response-prevention for obsessive compulsive disorder in childhood and adolescence J Behav Ther Exp Psychiatry 2008 7 11 22 10.1016/j.jbtep.2006.11.002 17207457

[B68] Storch EA Geffken GR Merlo LJ Mann G Duke D Munson M Family-based cognitive-behavioral therapy for pediatric obsessive-compulsive disorder: comparison of intensive and weekly approaches J Am Acad Child Adolesc Psychiatry 2007 7 469 478 10.1097/chi.0b013e31803062e7 17420681

[B69] Riddle MA Reeve EA Yaryura-Tobias JA Yang HM Claghorn JL Gaffney G Fluvoxamine for children and adolescents with obsessive-compulsive disorder: a randomized, controlled, multicenter trial J Am Acad Child Adolesc Psychiatry 2001 7 222 229 10.1097/00004583-200102000-00017 11211371

[B70] Geller DA Hoog SL Heiligenstein JH Ricardi RK Tamura R Kluszynski S Fluoxetine treatment of obsessive-compulsive disorder in children and adolescents: a placebo-controlled clinical trial J Am Acad Child Adolesc Psychiatry 2001 7 773 779 10.1097/00004583-200107000-00011 11437015

[B71] Storch EA Lewin AB de Nadai AS Murphy TK Defining treatment response and remission in obsessive-compulsive disorder: a signal detection analysis of the children's yale-brown obsessive compulsive scale J Am Acad Child Adolesc Psychiatry 2010 7 708 717 20610140 10.1016/j.jaac.2010.04.005

[B72] Tolin DF Is cognitive-behavioral therapy more effective than other therapies? A meta-ananlytic review Clin Psychol Rev 2010 7 710 720 10.1016/j.cpr.2010.05.003 20547435

[B73] Skoog G Skoog I A 40-year follow-up of patients with obsessive-compulsive disorder Arch Gen Psychiatry 1999 7 121 127 10.1001/archpsyc.56.2.121 10025435

[B74] Thomsen PH Children and adolescents with obsessive-compulsive disorder. A 6–22 year follow-up study of social outcome Eur Child Adolesc Psychiatry 1995 7 112 122 10.1007/BF01977739 7796249

